# Genetic adult lactase persistence is associated with risk of Crohn's Disease in a New Zealand population

**DOI:** 10.1186/1756-0500-3-339

**Published:** 2010-12-19

**Authors:** Deborah J Nolan, Dug Yeo Han, Wen Jiun Lam, Angharad R Morgan, Alan G Fraser, Linda C Tapsell, Lynnette R Ferguson

**Affiliations:** 1 Smart Foods Centre, University of Wollongong, Northfields Ave, Wollongong, New South Wales, Australia; 2 Nutrigenomics New Zealand, Discipline of Nutrition, University of Auckland, Park Rd, Auckland, New Zealand; 3 Discipline of Nutrition, University of Auckland, Park Rd, Auckland, New Zealand; 4 Illawarra Health and Medical Research Institute, University of Wollongong, Northfields Ave, Wollongong, New South Wales, Australia

## Abstract

**Background:**

*Mycobacterium avium *subspecies *paratuberculosis *(MAP) is an infective agent found in ruminants and milk products, which has been suggested to increase the risk of gastrointestinal inflammation in genetically susceptible hosts. It is hypothesized that lactase persistence facilitates exposure to such milk products increasing the likelihood of adverse outcomes. Individuals either homozygous or heterozygous for the T allele of DNA variant, rs4988235, located 14kb upstream from the *LCT *locus, are associated with having lactase persistence.

The aim of this study was to determine whether lactase persistence as evident by the T allele of rs4988235 is associated with Crohn's Disease (CD) in a New Zealand population.

**Findings:**

Individuals homozygous for the T allele (T/T genotype) showed a significantly increased risk of having CD as compared with those homozygous for the C allele (OR = 1.61, 95% CI = 1.03-2.51). Additionally, a significant increase in the frequency of the T allele was observed in CD patients (OR = 1.30, 95% CI = 1.05-1.61, p = 0.013), indicating that the T allele encoding lactase persistence was associated with an increased risk of CD.

**Conclusions:**

Our findings indicate that lactase persistence as evident by the presence of the T allele of rs4988235 is associated with risk of CD in this New Zealand Caucasian population.

## Background

Crohn's Disease (CD) is a form of inflammatory bowel disease of both genetic and environmental aetiology. Somewhat controversially *Mycobacterium avium *subsp. *Paratuberculosis *(MAP) exposure has resurfaced within the literature as a potential environmental variant that may be implicated in the development of CD [1]. MAP is an infective agent frequently found in ruminants such as cows and sheep that results in Johne's disease, an inflammatory bowel condition similar to that experienced by humans with CD [2]. MAP is secreted into the milk of infected ruminants [3] and is delivered to humans via the consumption of milk products [4] due to its reported resistance to commercial pasteurisation methods [5,6].

One of the most important factors that may influence the amount of milk consumed, and hence exposure to MAP in humans relates to the presence of Lactase-Phlorizin Hydrolase (lactase) in the small intestinal mucosa. Lactase is the enzyme required to hydrolyse lactose, the sugar found in milk products, into its constituent galactose and glucose [7]. Infants are born with the ability to digest lactose, but for the majority of the world's population this declines with age, a phenomenon known as lactase non persistence. However, lactase persistence is highly prevalent in descendants from populations with a rich pastoralist heritage [8]. Greater than 70% of individuals of white European heritage are reportedly lactose persistent compared to less than 40% throughout individuals from Asian countries [9].

Ennatah et al [10] identified a DNA variant, rs4988235 (c.1993+327 C > T, previously termed C/T-13910); 14kb upstream from the *LCT *locus that demonstrated complete agreement with clinically identified lactase non persistence in a Finnish population. *LCT *is a gene which encodes Lactase Phlorizin Hydrolase (LPH) for the hydrolysis of lactose. Homozygotes for the C allele of rs4988235 were found to be lactase-non persistent, whilst those heterozygous or homozygous for the T allele were determined to be lactase persistent. This finding has been replicated in several other European populations [11,12] and support for the functionality of this DNA variant has been demonstrated *in vitro *[13]. Thus the C/C genotype for rs4988235 is now a widely accepted marker of adult-type hypolactasia in Europeans.

Evaluating the association of lactase persistence with CD as a key factor influencing exposure to MAP via the consumption of milk products is particularly important in New Zealand, a country with one of the highest incidences of CD in the Western world at 16.5/100,000 per year [14] coupled with the highest herd prevalence estimate of Johne's disease at 60% [5].

The aim of this study was to determine whether lactase persistence as evident by the presence of the T allele of rs4988235 is associated with CD in a New Zealand population.

## Methods

### Samples

The Auckland CD Project is a population-based study of genetic and environmental determinants of CD aetiology. CD patients were recruited between May 2005 and April 2007 through local doctors and surgeries in Auckland, New Zealand, and also other North Island centres, by media campaigns, e.g. local newspaper and television. Controls were recruited by the same means, and also recruited from non-affected family members and friends of CD patients. Participants consented to collection of peripheral blood for DNA extraction and genotyping.

A total of 945 subjects (333 CD patients and 612 controls) consented to take part. The cases in this study are a subset of the Caucasian participants of the Auckland CD Project. Their gastroenterologists were approached, clinical records scrutinised to confirm diagnosis, and CD was defined using standard diagnostic criteria [15]. Cases were phenotyped according to the Montreal Classification systems, allowing genotype-phenotype analysis to be performed. Phenotypic information was available for 323 CD patients. All participants included in this study were of self-reported European ancestry (subjects who self-reported having any Maori or other non-Caucasian ancestry are not included in the dataset).

The study was conducted under ethical protocol MEC/04/12/011, authorised through the New Zealand Multi-Region Human Ethics Committee. All study subjects gave informed consent.

DNA was extracted from blood samples or buccal swabs using Qiagen's DNA extraction kit (Qiagen, Valencia, CA, USA) and following the manufacturer's instructions.

### Genotyping

Genotyping of rs4988235 was performed using the ABI TaqMan MGB diallelic discrimination system with forward and reverse primer sequences 5'-CTG CGC TGG CAA TAC AGA TAA G-3' and 5'AAA TGC AAC CTA AGG AGG AGA GTT C-3' respectively and VIC-- ATA ATG TAG TCC CTG GCC T and FAM--ATA ATG TAG CCC CTG GC probes (Applied Biosystems, Melbourne, Australia). The reactions were prepared using 2× TaqMan Universal Master Mix, 40× SNP Genotyping Assay Mix, DNase-free water, and 10 ng genomic DNA in a final volume of 5 ul per reaction. The PCR amplification was performed using the ABI Prism 7900 HT sequence-detector machine under the following conditions: 50°C for 2 minutes, 10 min 95°C enzyme activation followed by 40 cycles at 95°C for 15 sec and 60°C for 1 min (annealing/extension).

To reduce the possibility of genotyping error, genotyping was repeated utilising different primers (5'-CTC TGC GCT GGC AAT ACA G and 5'-AAA TGC AAC CTA AGG AGG AGA) and probes (VIC-ATA AGA TAA TGT AGC CCC TGG C and FAM- ATA AGA TAA TGT AGT CCC TGG C) (Applied Biosystems, Melbourne, Australia) under the following conditions: 60°C for 1 minute, 95°C for 10 minutes, followed by 55 cycles of 92°C for 15 s and 60°C for 1 minute and 60°C for 1 minute.

The allelic discrimination results for both sets of genotyping were determined after the amplification by performing an endpoint read.

Assays were optimized in 24 samples consisting of 20 reference Centre d'Etude du Polymorphisme Humain (CEPH) samples and 4 blanks. All sample plates contained cases, controls, blanks, CEPH and duplicate samples. Quality control measures included independent double genotyping, blind to sample identity and blind to the other caller, and comparison of our CEPH genotypes to those in the HapMap http://www.hapmap.org.

### Statistical analysis

The allelic trend test [16] was used to compare frequencies between case and control alleles. An exact test was used to test for departures from Hardy-Weinberg equilibrium (HWE) in the case and the control samples [17]. Lactase persistence was defined as having genotypes C/T and T/T of rs4988325 whilst C/C was defined as lactase non-persistence.

Genotypic analysis was carried out and the allelic odds ratio with confidence interval was calculated to test the effect of the T allele on CD risk.

SAS (V9.1 SAS Institute., Cary, NC, USA) and R (Ihaka and Gentleman, 1996) were used for statistical analyses.

## Results

The genotypes did not meet Hardy-Weinberg equilibrium (HWE) for both the cases and controls. To ensure genotyping errors did not exist in the data, genotyping was repeated using an alternative primer and probe set and the genotypes were confirmed to be correct. Also, the minor allele frequency was 0.273 which is similar to the HapMap CEU population frequency of 0.274. Deviations from HWE may have resulted by chance, as a result of population admixture or perhaps the rs4988235 SNP is associated with a currently unknown factor in controls.

For rs4988235, the T/T genotype showed a significantly increased risk of having CD as compared with the C/C genotype (OR = 1.61, 95% CI = 1.03-2.51) however, no significant difference was found between C/T and C/C. Additionally, a significant increase in the frequency of the T allele was observed in CD patients (OR = 1.30, 95% CI = 1.05-1.61, p = 0.013), indicating that the T allele encoding lactase persistence was associated with risk of CD (Table 1).

**Table 1:** Genotype and allele count frequencies for rs4988235 in CD patients and in controls

Genotype Allele	CD N (%)	Control N(%)	OR (95%CI)	p
C/C	32 (9.6)	82 (13.4)	1.00	0.065

C/T	118 (35.4)	238 (38.9)	1.27 (0.80-2.02)	

T/T	183 (55.0)	292 (47.7)	**1.61 (1.03-2.51)**	

Z = 2.34, p < Z 0.009*				

T	484 (72.7)	822 (67.2)	**1.30 (1.05-1.61)**	**0.013**

C	182 (27.3)	402 (32.8)	1.00	

*Cochran-Armitage Trend Test

Genotype-phenotype analysis: of 323 cases, 88 (28.4%) had relatives with IBD, 98 (31.6%) had undergone a bowel resection, 44 (14.2%) had extra-intestinal manifestations of CD occurring and 112 (46.9%) smoked at diagnosis (Table 2).

**Table 2:** Summary of clinical data in subgroups of CD patients with a T allele for rs4988235

	N (%)	OR (95% CI)	p
Age at first diagnosis			
0-16 years	30 (10.6)	0.98 (0.55-1.75)	1.00
17-40 years	203 (71.5)	**1.36 (1.05-1.76)**	**0.021**
> 40 years	51 (18.0)	1.56 (0.96-2.53)	0.087
CD location			
Ileal	97 (36.5)	**1.50 (1.05-2.15)**	**0.032**
Colonic	86 (32.3)	1.26 (0.88-1.81)	0.245
Ileocolonic	83 (31.2)	1.25 (0.86-1.81)	0.273
CD Behaviour			
Inflammatory	152 (57.6)	**1.36 (1.02-1.82)**	**0.039**
Stricturing	81 (30.7)	1.27 (0.87-1.85)	0.233
Penetrating	31 (11.7)	1.41 (0.77-2.57)	0.315
Relative with IBD	88 (28.4)	**1.71 (1.17-2.51)**	**0.006**
Bowel resection	98 (31.6)	**1.47 (1.03-2.10)**	**0.033**
Extra Intestinal Manifestations	44 (14.2)	1.38 (0.84-2.28)	0.228
Smoker at diagnosis	CD: 112 (46.9) Control: 130 (31.7)	1.04 (0.69-1.56)	0.918

*IBD - Inflammatory Bowel Disease

Individuals carrying the T allele of rs4988235 had an increased probability of developing CD that was diagnosed between 17 and 40 years (OR = 1.36, 95% CI = 1.05-1.76, p = 0.021); and a significantly increased risk of developing ileal CD (OR = 1.50, 95% CI = 1.05-2.15, p = 0.032) or CD of an inflammatory nature (OR = 1.36, 95% CI = 1.02-1.82, p = 0.039). In addition, a significantly increased risk of having other relatives with IBD and of undergoing bowel resection was observed in individuals with the T allele of rs4988235 (OR = 1.71, 95% CI = 1.17-2.51, p = 0.006 and OR = 1.47, 95% CI = 1.03-2.10, p = 0.033 respectively).

## Discussion

Results from this study suggest that genetic adult-type lactase persistence as evident by the T allele of rs4988235 may increase the risk of developing CD in this New Zealand population.

Our findings are in agreement with Juste et al [18], who reported an increased frequency of the T allele of rs4988235 in CD patients (61.9%) compared with controls (47.1%, p = 0.0275). Similarly a highly significant correlation between incidence of CD and lactase persistence (r = -0.655, p = 0.0017) was reported in an observational study encompassing data from 20 countries [19], whilst Shrier et al [20] reported a reduction in CD risk with increasing prevalence of lactase non-persistence in a review of global population data.

Conversely, Büning et al [21] failed to find an association between the C/C genotype of rs4988235 and the pathogenesis of CD in a German case-control study of 166 CD patients and 187 healthy controls. This discrepancy may relate to the smaller sample size of this study; however evaluation of this phenomenon on a regional basis, taking into consideration adult lactase status, dairy food consumption, and degree and management of MAP infection within ruminant populations may provide evidence to ensure that a consensus can be reached regarding the role of lactase persistence and MAP exposure in the aetiology of CD.

The finding that lactase persistence was associated with an increased risk of having a relative with inflammatory bowel disease may reflect the autosomal dominant nature of lactase persistence, whilst the increased risk of CD diagnosis between 17-40 years of age may correspond with the onset of hypolactasia that typically occurs in adulthood in susceptible individuals. The T allele for rs4988235 was also associated with an increased risk of inflammation, having an ileal CD location and bowel resection amongst CD cases. This was an unexpected finding and warrants further investigation to conclusively determine whether lactase persistence may influence the phenotypic expression of CD.

As associations between MAP and inflammatory bowel disease have largely been limited to individuals with CD, this study did not evaluate lactase persistence in the ulcerative colitis context. However, the inclusion of this clinical population in future research may provide further insights into the potential association between lactase persistence and inflammatory bowel disease.

Whether lactase persistence is an independent risk factor for CD or merely a marker of the likelihood of exposure to the MAP pathogen, found predominantly in milk products, is beyond the scope of this particular study. However epidemiological evidence is conflicting, indicating an urgent need for further, well controlled dietary and genetic case-control studies to distinguish between these two hypotheses.

In Japan, Shoda et al [22] reported that incidence of CD was strongly correlated with consumption of milk protein and Shrier et al [18] identified a trend with estimated yearly per capita dairy food consumption and increased risk of CD. In contrast Abubakar et al [23] reported that consumption of pasteurized milk was associated with a decreased risk of CD in a case-control study of 218 individuals with CD in England.

Recent advances in technology have ensured that the presence of MAP *in vivo *is more accurately detected [24]. These advances have contributed to the resurgence of the MAP and CD hypothesis. Viable MAP was recently identified in the peripheral blood of a high proportion of individuals with CD, with only MAP DNA isolated in a small proportion of controls [25]. This may reflect different physiological responses to the pathogen between cases and controls. These findings were supported locally in a New Zealand study, utilising a larger cohort of 361 CD patients and 200 controls. In this study MAP DNA in peripheral blood was found to be over-represented in CD patients in comparison to controls [26].

The Zoonotic potential of MAP was discussed in a review by Grant [5], who reported that hypotheses suggesting a causal role for MAP in the pathogenesis of CD were plausible, however insufficient available evidence can either prove or disprove this idea at present.

Results from this exploratory study do not prove that MAP exposure is responsible for the development of CD; or that milk products increase risk of the development of CD. However our findings indicate that there may be a complex interaction between exposure to this infectious agent as a consequence of an evolutionary adaptation to ensure tolerance to the milk products that may serve as the medium for its delivery to humans. As the worldwide incidence of CD is rapidly increasing, [27-29], there is an urgent need to determine whether our findings can be replicated in larger populations. If so, efforts to eradicate MAP through animal vaccination and greater prevention of its contamination into the food supply should become key public health priorities.

## Conclusion

Following a case-control evaluation of rs4988235 in a New Zealand CD population, we found that lactase persistence as evident by the presence of a T allele was associated with increased CD risk. This finding may relate to the increased exposure to dairy products in individuals with lactase persistence, a food group that has been implicated in the aetiology of CD due to the presence of MAP species in milk. Further research is required to substantiate our findings and to conclusively determine the nature of the observed association between lactase persistence and CD.

## Competing interests

The authors declare that they have no competing interests.

## Authors' contributions

DJN conceived the study and conducted the genotyping of blood samples with WJL. DJN assisted with data analysis, interpretation of data and drafted the manuscript. DYH performed the statistical analyses and contributed to the manuscript. ARM made suggestions to the study and contributed to the manuscript. AGF provided the clinical data and many of the samples obtained. LRF made suggestions for the study and revised the manuscript. LCT revised the manuscript. All authors read and approved the final draft.
